# Contribution of Proteins and Peptides to the Impact of a Soy Protein Isolate on Oxidative Stress and Inflammation-Associated Biomarkers in an Innate Immune Cell Model

**DOI:** 10.3390/plants12102011

**Published:** 2023-05-17

**Authors:** Giselle Franca-Oliveira, Adolfo J. Martinez-Rodriguez, Esperanza Morato, Blanca Hernández-Ledesma

**Affiliations:** 1 Institute of Food Science Research (CIAL, CSIC-UAM, CEI UAM+CSIC), Nicolás Cabrera, 28049 Madrid, Spain; g.f.oliveira@csic.es; 2 Center of Molecular Biology “Severo Ochoa” (CBMSO), CSIC-UAM, Nicolás Cabrera 1, 28049 Madrid, Spain; emorato@cbm.csic.es

**Keywords:** soybean protein isolate, antioxidant activity, lunasin, immune cells, oxidative-stress-associated biomarkers

## Abstract

The innate and adaptative immune systems are involved in the regulation of inflammatory and oxidative processes and mediators such as reactive oxygen species (ROS) and nitric oxide (NO). The exacerbated action of these players results in an oxidative stress status and chronic inflammation, which is responsible for the development of non-communicable diseases (NCDs). By modulating these mediators, bioactive compounds in food can exert a key role in the prevention of several NCDs. Among these compounds, soybean proteins and peptides such as lunasin have been considered to be among the most promising. The aim of this study was to obtain and characterize a soluble protein-enriched extract from a commercial soybean protein isolate and fractionate it into different fractions through ultrafiltration. Their antioxidant and immunomodulatory properties were then evaluated using biochemical and cell models. A total of 535 proteins (from 282 protein groups) were identified in the extract, in which the presence of the peptide lunasin was confirmed. The enrichment of this peptide was achieved in the 3–10 kDa fraction. The protective effects against the oxidative stress induced by LPS in the macrophage model could have been mediated by the radical scavenging capacity of the peptides present in the soybean samples. Under basal conditions, the extract and its ultrafiltered fractions activated macrophages and induced the release of NO. However, under challenged conditions, the whole extract potentiated the NO-stimulating effects of LPS, whereas the fraction containing 3–10 kDa peptides, including lunasin, counteracted the LPS-induced NO increase. Our findings suggest a promising role of soybean protein as an ingredient for functional foods and nutraceuticals aimed at promoting health and preventing oxidative stress and/or immune-alteration-associated diseases.

## 1. Introduction

Non-communicable diseases (NCDs) affect a high percentage of the world’s population and are the most important cause of death and disability in the world. Some of the most prevalent include cardiovascular and neurological diseases, obesity and diabetes, inflammatory bowel diseases, and cancer [1]. These disorders are associated with an altered immune response initiated in the tissues and organs due to multiple and combined lifestyle factors [2,3]. The immune response is a complex process of cell activation by the recognition of patterns associated with pathogens or endogenous stress signals, resulting in an acute inflammatory state aimed at eliminating the threat, repairing the damage, and recovering the tissues [4]. The cellular stimulation of inflammatory processes is triggered by cytokines and chemokines, followed by the coordination of mechanisms in which other mediators such as reactive oxygen species (ROS) and nitric oxide (NO) can play key roles [5]. The action of all these players results in an oxidative stress status and prolonged inflammation known as systemic chronic inflammation, which has been associated with health impairment and the development of NCDs [6]. Environmental conditions and, in particular, the diet have been recognized as the main factors behind most of these diseases [7]. Thus, while the overconsumption of processed foods and beverages has been associated with an increased risk of NCDs, the intake of foods containing bioactive compounds has been recognized to reduce or even prevent several of these disorders [8,9]. There is increasing evidence on the beneficial role that these compounds can exert on oxidative stress and inflammation biomarkers, making them promising alternatives to protect against the associated diseases. Among these compounds, those found in vegetal sources are attracting special interest. Soybean (*Glycine max*) is a legume of the Fabaceae family, which is native to the Asian continent, whose market has acquired great importance, becoming one of the most cultivated and traded vegetables in the world. Because of its high content and quality of proteins (≈42%) and lipids (≈22%), soybean has a broad range of uses from livestock feed to industrial purposes. In Asia, the consumption of soybean is mostly in fermented or simply cooked forms, such as edamame, soy milk, tofu, and tempeh [10,11]. However, in Western countries, soybean is mainly processed into flour-derived products such as texturized protein and protein concentrates or isolates, which are added to different food products [12,13]. Besides its high nutritional value, the consumption of soybean has been associated with low incidence and prevalence of several NCDs [14]. It has been described that its consumption has positive impacts on different pathologies such as metabolic and cardiovascular diseases, bone health, some types of cancer, kidney function, the symptoms of menopause, cognitive functions, mental health, skin health, and fertility. These beneficial effects have been attributed to proteins, lipids, carbohydrates, and other minor components such as phytic acid, saponins, and isoflavones [15,16].

In addition to its biological and functional properties and its value as a source of essential amino acids, soybean protein is considered a promising source of bioactive peptides. These peptides are inactive within the precursor protein, but once released by hydrolysis, digestion, or fermentation processes, they can exert different physiological effects [16]. Among soybean-derived peptides, lunasin has become one of the most studied because of its multifunctionality and its potential application in health promotion. This peptide, identified in the soybean cotyledon [17,18], contains 43 amino acids in its monomeric form. Its sequence is SKWQHQQDSCRKQLQGVNLTPCEKHIMEKIQGRGDDDDDDDDD, although some researchers have also reported a soybean-derived lunasin with an additional asparagine residue at the C-terminus [19]. Different properties have been attributed to lunasin such as antioxidant, anti-inflammatory, and hypocholesterolemic properties, among others [20]. In our previous study, lunasin was identified as one of the major peptides responsible for the effects of an albumin-enriched extract on different immune cell models [21]. However, in addition to lunasin, other peptides present in soybean have been reported to exert multiple health benefits such as anti-diabetic, angiotensin converting enzyme inhibitory, anti-hypertensive, phagocytosis-stimulatory, triglyceride-reducing, immunostimulatory, appetite-suppressing, proteinase-inhibitory, and chemopreventive properties, and contribute on the beneficial health attributed to this legume [22].

Bioactive peptide research is constantly advancing, identifying new sequences with distinct activities, and elucidating their mechanisms of action, thereby contributing to the development of new peptide-based ingredients for health-promoting foods and nutraceuticals [16,23]. Therefore, the aim of this study was to obtain and characterize a soluble protein-enriched extract from a commercial soybean protein isolate and fractionate it into different fractions through ultrafiltration. The potential of the proteins/peptides contained in the extract and the fractions as modulators of biomarkers associated with oxidative stress and an altered immune response was also evaluated using biochemical and cell models.

## 2. Materials and Methods

### 2.1. Materials

2,2′-Azino-bis (3-ethylbenzothiazoline-6-sulfonic acid) (ABTS), 6-hydroxy-2,5,7,8-tetramethylchromium-2-carboxylic acid (Trolox), fluorescein disodium (FL), 2,2′-azobis (2-amidinopropane dihydrochloride) (AAPH), 3-(4,5-dimethylthiazol-2-yl)-2,5-diphenyltetrazol bromide (MTT), dimethyl sulfoxide (DMSO), and 2′7′-dichlorofluorescein diacetate (DCFA-DA) were acquired from Sigma-Aldrich (St. Louis, MO, USA). Lipopolysaccharide (LPS) was purchased from Merck (Kenilworth, NJ, USA). All other reagents were of analytical grade.

The soybean protein isolate (PI) was provided by Emilio Peña Aditivos Alimentarios S.A. (Valencia, Spain). The lunasin peptide was synthesized by DGpeptides Co., Ltd. (Hangzhou, China) with a purity of 95.2%.

### 2.2. Preparation of a Lunasin-Enriched Soy Protein Isolate (LPI)

The lunasin-enriched soy protein isolate extract (LPI) was obtained from the PI via aqueous solvent extraction. First, 5 g of the PI was dissolved in 50 mL of 0.1 M PBS (pH 7.4) by stirring the solution at room temperature for 3 h. After sonicating the mixture for 1 h and centrifugation at 12,000× *g* for 30 min at 4 °C, the supernatant was collected via filtration, lyophilized, and kept (LPI) at −20 °C until further assays. The protein concentration of the LPI was determined using the Kjeldahl method, applying a conversion factor of 5.71 [24].

Three peptide fractions (>10 kDa, LPI-UF1), (3–10 kDa, LPI-UF2), and (<3 kDa, LPI-UF3) were obtained via ultrafiltration using Amicon^®^ Ultra−15, 10 K Millipore^®^ (Burlington, MA, USA), and Vivaspin^®^ 20 mL Sartorius^®^ (Gottingen, Germany) devices, following the manufacturers’ instructions. The protein concentrations of the fractions were determined via a bicinchoninic acid (BCA) assay using a Pierce BCA kit (Thermo Scientific, Waltham, MA, USA). Bovine serum albumin (BSA) was used as a standard.

### 2.3. Proteomic Analysis

The proteomic analysis of the PI and LPI was carried out at the “Center of Molecular Biology Severo Ochoa (CBMSO) Protein Chemistry Facility” (CSIC, Madrid, Spain). Both samples were suspended in 40 µL of sample buffer (corresponding to 25 µg of protein) and loaded onto 1.2 cm wide wells of a conventional sodium dodecyl sulfate–polyacrylamide gel electrophoresis (SDS-PAGE) gel (0.75 mm thick, 4% stacking, and 10% resolving; Bio-Rad Laboratories, Madrid, Spain). Once the front entered 3 mm into the gel, the run was stopped. After visualizing the unseparated protein bands using Coomassie staining, they were excised, cut into 2 × 2 mm cubes, and placed in microcentrifuge tubes [25]. An acetonitrile/water (1:1) solution was used to destain the gel pieces that were further reduced with 10 mM dithiothreitol (DTT) for 1 h at 56 °C, alkylated with 10 mM iodoacetamide for 30 min at room temperature in darkness, and digested with sequencing-grade trypsin (Promega, Madison, WI, USA) and chymotrypsin (Roche, Mannheim, Germany) as previously described [26], with minor modifications. The gel pieces were shrunk using acetonitrile and dried in a speedvac. The dried gel pieces were re-swollen in 100 mM Tris-HCl (pH 8.0) and 10 mM CaCl_2_ with 60 ng/µL trypsin or chymotrypsin at a 5:1 protein/enzyme (*w*/*w*) ratio. The tubes were kept on ice for 2 h and incubated at 37 °C (trypsin) or 25 °C (chymotrypsin) for 12 h. Then, 1% trifluoroacetic acid was used to stop the digestion. Whole supernatants were dried and desalted onto ZipTip C18 pipette tips (Merk KGaA, Darmstadt, Germany) until the mass spectrometric analysis.

The dried desalted protein digest was dissolved in 10 µL of 0.1% formic acid (2 µg of digested peptide) and analyzed using reverse-phase liquid chromatography coupled to tandem mass spectrometry (RP-LC-MS/MS) in an Easy-nLC II system coupled to an ion trap LTQ-Orbitrap-Velos-Pro hybrid mass spectrometer (Thermo Scientific). The peptides were concentrated (on-line) via RP using a 0.1 mm × 20 mm C18 RP precolumn (Thermo Scientific) and then separated using a 0.075 mm × 250 mm C18 RP column (Phenomenex, Torrance, CA, USA) operating at 0.22 μL/min. Peptides were eluted using a 60 min dual gradient. The gradient profile was set as follows: 5–25% solvent B for 45 min, 25–40% solvent B for 15 min, 40–100% solvent B for 2 min, and 100% solvent B for 18 min (Solvent A: 0.1% formic acid in water, solvent B: 0.1% formic acid and 80% acetonitrile in water). ESI ionization was performed using a Nano-bore emitters Stainless Steel ID 30 μm (Thermo Scientific) interface at a spray voltage of 2.1 kV with an S-lens of 60%. The orbitrap resolution was set to 30,000 [27]. Peptides were detected in survey scans from 400 to 1600 amu (1 μscan), followed by twenty data-dependent MS/MS scans (top 20), using an isolation width of 2 u (in mass-to-charge ratio units), a normalized collision energy of 35%, and dynamic exclusion applied for 60 s periods. Charge-state screening was enabled to reject unassigned and singly charged protonated ions.

Peptide identification was carried out using the PEAKS Studio XPro search engine (Bioinformatics Solutions Inc., Waterloo, ON, Canada). A database search was performed against Uniprot-Soybean, (74863 entries; UniProt release 12/20) (decoy-fusion database). The following constraints were used for the searches: tryptic cleavage after arginine and lysine or chymotryptic cleavage after tyrosine, tryptophan, phenylalanine, and leucine (semi-specific), up to two missed cleavage sites, and tolerances of 20 ppm for precursor ions and 0.6 Da for MS/MS fragment ions, and the searches were performed while allowing optional methionine oxidation and cysteine carbamidomethylation. The false discovery rates (FDR) for peptide spectrum matches (PSMs) and protein were limited to 0.01. Only those proteins with at least two unique peptides being discovered in the LC/MS/MS analyses were considered reliably identified [28].

### 2.4. Preparation and Characterization of LPI and Ultrafiltered Fractions

#### 2.4.1. Gel Electrophoresis (SDS-PAGE)

The protein profiles of LPI and its fractions were analyzed using SDS-PAGE in Criterion automated equipment (Bio-Rad Laboratories) using 12% Criterion™ TGX™ Precast Midi Protein Gels (Bio-Rad). Samples (1 mg of protein/mL) were dissolved in a sample buffer containing 0.05 M Tris-HCl (pH 6.8), 1.6% (p:v) SDS, 8% (v:v) glycerol, 0.02% (p:v) 2-bromophenol, and 2% (v:v) β-mercaptoethanol, heated for 5 min at 100 °C, and cooled to room temperature. A Precision Plus Protein™ Dual Xtra Prestained Protein Standard (Bio-Rad Laboratories) was used as a molecular weight marker. Next, 40 µL of the sample was loaded onto the gel and run with voltages of 100 V (5 min) and 150 V (50 min). The commercial buffer XT MES Running Buffer 20X (Bio-Rad Laboratories) was used for the separation. The gel was stained with InstantBlue^®^ Commassie Protein Stain (Abcam, Cambridge, UK). A gel image was taken using the Versadoc Imaging System gel reader and analyzed using software image lab 6.1 (Bio-Rad Laboratories).

#### 2.4.2. Western Blot

The presence of lunasin in the LPI and its fractions was determined via Western blot, using synthetic lunasin as a standard (12.5–100 µM). Samples were loaded onto 16.5% Bis-Tris Criterion™ XT Precast gels (Bio-Rad Laboratories), and the electrophoretic migration was carried out at 60 V for 2 min, followed by 100 V for 3 h. Proteins and peptides were transferred to polyvinylidene membranes (PVDF) (Bio-Rad Laboratories) using a voltage of 18 V for 30 min. After blocking and washing the membrane, it was incubated overnight at 4 °C with the primary rabbit antibody against lunasin (Biomedal, Seville, Spain, 1:12,000). After washing the membrane, it was incubated for 1 h at room temperature with the IgG-horseradish peroxidase (HRP)-conjugated anti-rabbit IgG secondary antibody (Santa Cruz Biotechnology, Dallas, TX, USA; 1:5000). The ECL™ Prime Western Blotting Detection Reagent (Cytiva Amersham™, Buckinghamshire, UK) was used to develop the membrane. Images were taken on a Molecular Imager^®^ Versadoc™ MP 4000 using an AF 50 mm f/1 4D photographic objective (Nikon, Tokyo, Japan) and were analyzed using the software Quantity One 1-D (Bio-Rad Laboratories).

### 2.5. Biological Activity of the Lunasin-Enriched Soy Protein Isolate (LPI) and Its Fractions

#### 2.5.1. In Vitro Antioxidant Activity

The ABTS^•+^ scavenging activity was determined according to the enhanced protocol described by Re et al. [29]. A volume of 180 μL of a diluted ABTS^•+^ solution (formed by incubating 7 mM ABTS and 2.45 mM potassium persulfate in the dark overnight) and 20 μL of PBS (blank), Trolox (25–200 µM) (standard), or the sample (different concentrations) were mixed and incubated for 7 min at room temperature, and the absorbance was read at 734 nm in a Biotek SynergyTM HT plate reader (Winooski, VT, USA).

The oxygen radical absorbance capacity (ORAC) was measured following the protocol reported by Hernández-Ledesma and coworkers [30]. FL (117 nM), AAPH (14 mM), and either antioxidant (Trolox (0.2–1.6 nmol) or the sample (at different concentrations)) prepared in a 75 mM PBS buffer (pH 7.4) were mixed and incubated at 37 °C, recording the fluorescence every 2 min for 120 min at λ_excitation_ and λ_emmission_ values of 485 and 520 nm, respectively, in a Fluostar Optima BMG Labtech plate reader (Ortenberg, Germany). The equipment was controlled by the FLUOstar Control ver. 1.32 R2 software for fluorescence measurement. Both the TEAC and ORAC values were expressed as µmol Trolox equivalent (TE)/mg of protein.

#### 2.5.2. Effects in RAW 264.7 Macrophages

RAW 264.7 mouse macrophages (American Type Culture Collection, ATCC, Rockville, MD, USA) were grown in modified Dulbecco Eagle medium (DMEM) that was high in glucose (Biowest, Riverside, MO, USA) and supplemented with 10% fetal bovine serum (FBS) (*v*/*v*) and 1% penicillin/streptomycin/amphotericin (*v*/*v*) (Biowest). Cells were grown at 37 °C under constant conditions of humidity, 5% CO_2_, and 95% air.

##### Effects on Cell Viability

The evaluation of the effect of the LPI on cell viability was carried out using the MTT assay, following the reported protocol [31]. Briefly, cells were seeded onto 96-well plates (Sarstedt AG & Co., Nümbrecht, Germany) at a density of 1 × 10^6^ cells/mL and incubated at 37 °C for 24 h. After removing the culture medium, the LPI and its fractions dissolved in DMEM without FBS (0.25 to 1 mg of protein/mL) were added, and the plate was incubated for 24 h at 37 °C. DMEM without FBS was used as a negative control. After discarding the treatment, MTT solution (0.5 mg/mL) was added, and cells were incubated for 2 h at 37 °C. The formazan crystals were dissolved in DMSO, and the absorbance was measured at 570 nm in a Multiskan FC plate reader (Thermo^TM^ Scientific, Wilmington, DE, USA). The results were expressed as a percentage of the control, which was considered as 100%.

##### Effects on Reactive Oxygen Species (ROS) Generation

The effects of LPI and its fractions on the intracellular ROS levels were determined using DCFH-DA as a fluorescent probe [32]. Cells were seeded (4.75 × 10^4^ cells/mL) onto a 48-well plate (Corning Costar Corp., Corning, NY, USA) and incubated at 37 °C for 24 h. After discarding the medium, 120 μL of LPI or its fractions at concentrations ranging from 1 mg of protein/mL to 0.25 mg of protein/mL were added, and the plate was incubated for 24 h at 37 °C. DMEM without FBS and DMEM + LPS (100 ng/mL) were used as negative and positive controls, respectively. Once removed from the treatment, cells were treated with DCFH-DA (0.4 mg/mL) dissolved in Hank’s Balanced Salt Solution (HBSS, Sigma-Aldrich) for 1 h at 37 °C, and the fluorescence was measured at the excitation and emission wavelengths of 485 nm and 520 nm, respectively, in a Fluostar Optima BMG Labtech plate reader (BMG Labtech). The results were expressed as ROS levels (% compared to the control, which was considered as 100%).

##### Effects on Nitric Oxide (NO) Levels

A Griess assay was conducted to determine the effects of LPI and its fractions on the release of NO. Cells were seeded (1 × 10^5^ cells/well) onto 96-well plates (Corning Costar Corp.) and incubated at 37 °C for 24 h. After aspirating the medium, cells were treated with LPI and its fractions at concentrations ranging from 1 to 0.25 mg of protein/mL for 24 h at 37 °C. The negative and positive controls were DMEM without FBS and DMEM + LPS (60 ng/mL), respectively. A volume of 100 µL of the supernatant was mixed with 100 µL of Griess reagent and incubated for 15 min at room temperature, measuring the absorbance at 540 nm in a Biotek SynergyTM HT reader. The amount of NO was calculated using a NaNO_2_ standard curve (3.125–100 μM).

### 2.6. Statistical Analysis

The obtained results were analyzed using a one-way ANOVA, followed by a Tukey test, using the statistical analysis program GraphPad Prism 8.0 (GraphPad Software, San Diego, CA, USA).

## 3. Results and Discussion

### 3.1. Characterization of the Lunasin-Enriched Soybean Protein Isolate Extract (LPI) and Its Fractions

Table 1 shows the protein contents of PI, LPI, and its ultrafiltered fractions. The content was reduced from 75.59% (PI) to 35.49% (LPI) when the aqueous solvent-soluble proteins/peptides were extracted with PBS. After ultrafiltration, the protein contents of the fractions were 33.99, 3.10, and 1.30% for LPI-UF1, LPI-UF2, and LPI-UF3, respectively. This result indicated that LPI, as expected, was mainly composed of high-molecular-weight proteins, although smaller proteins and peptides were also present in LPI, which could contribute to its potential biological activities.

**Figure 1:**
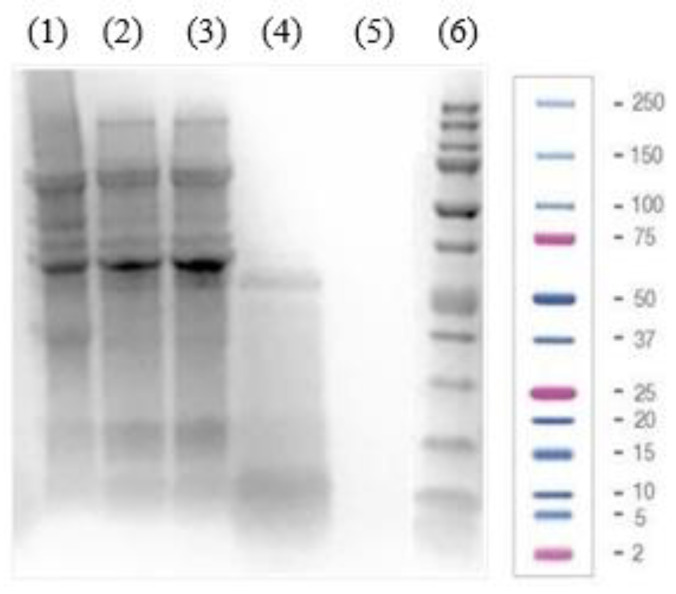
Electrophoretic (SDS-PAGE) analysis of (1) soy protein isolate (PI, 40 µg of protein), (2) lunasin-enriched PI (LPI, 40 µg of protein), and its ultrafiltered fractions ((3) the fraction higher than 10 kDa (LPI-UF1, 40 µg of protein), (4) the 3–10 kDa fraction (LPI-UF2, 40 µg of protein), (5) the fraction lower than 3 kDa (LPI-UF3, 40 µg of protein)) as well as (6) a Precision Plus Protein™ Dual Xtra Prestained Protein Standard.

The protein profiles of PI, LPI, and its fractions were analyzed using SDS-PAGE. As shown in Figure 1, bands ranging from 4 to 138 kDa were detected for PI, LPI, and LPI-UF1, identifying the major soybean proteins 7S β-conglycinin, 11S glycinin, and 2S albumin, with molecular weights between 52 and 72 kDa, between 20 and 43 kDa, and <25 kDa, respectively [15,33].

After removing the insoluble proteins, the bands disappeared in the lane corresponding to the LPI, while the intensity of highly soluble proteins increased in comparison to those appearing in the lane corresponding to the PI. The fractionation through ultrafiltration enriched the fractions according to their molecular weights. Thus, bands corresponding to proteins higher than 10 kDa showed higher intensity in LPI-UF1, while the enrichment of proteins/peptides with molecular weights ranging from 3 to 10 kDa was notable in LPI-UF2. Due to the characteristics of the gel and the electrophoresis conditions, no bands were visible for LPI-UF3, which contained small peptides and free amino acids.

**Figure 2:**
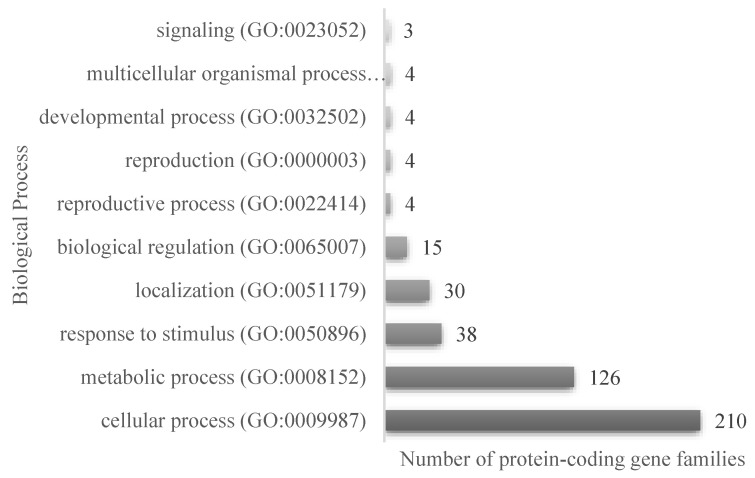
Classification of the proteins identified in the LPI proteomic analysis, according to the annotated biological process in PantherGO-Slim, for the 535 protein accessions.

The results of the proteomic analysis of both the PI and LPI samples are shown in Supplementary Tables S1 and S2, respectively. A total of 535 protein sequences were detected in LPI, with an average mass between 9 and 100 kDa (Supplementary Table S2), corresponding to the main proteins present in soybean. Enzyme inhibitors such as the Bowman–Birk protease inhibitor (BBI) and the Kunitz trypsin inhibitor (KTI) were also identified. These inhibitors have been reported to protect the peptide lunasin from the action of digestive enzymes during transit through the gastrointestinal tract [34]. The Panther database was used to classify the identified sequences [35]. In total, 429 protein-coding gene families were associated with annotated biological processes, of which 210 (49.0%) corresponded to proteins participating in cellular processes and 126 sequences corresponded to proteins implicated in metabolic processes (Figure 2). The sequences were also classified according to the cellular components annotated in the mentioned database. It was found that 25.2% (108 sequences) of the classified proteins were related to protein-containing complexes, and 55.9% (240) were related to cellular anatomical entities.

**Figure 3:**
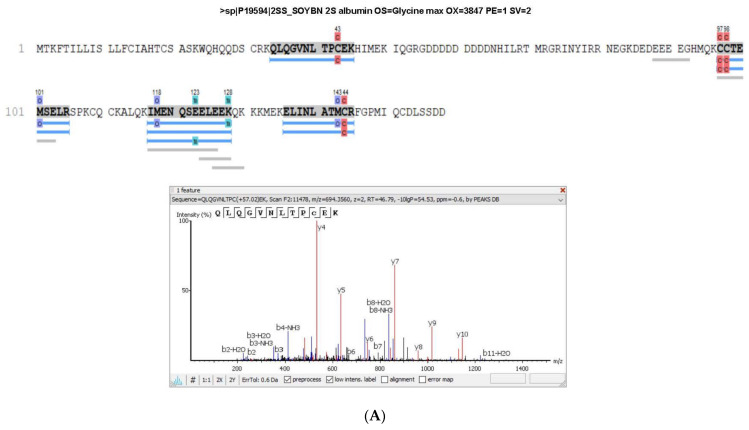

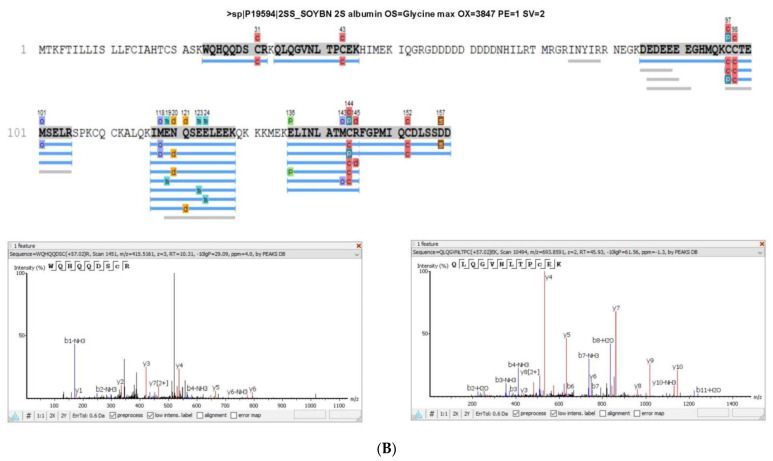
Sequence coverage in (**A**) soy protein isolate (PI) and (**B**) lunasin-enriched PI (LPI) for the 2S albumin protein. The spectra of the peptides WQHQQDS and QLQGVNLTPCEK, which correspond to the lunasin region, are shown.

The albumin 2S, in which the peptide lunasin is inserted, was also identified in the proteomic analysis of both PI and LPI (Figure 3), with a higher coverage for LPI than for PI. The fragment ^12^QLQGVNLTPCEK^25^ included in the lunasin sequence was detected in PI, while this fragment and the peptide ^2^WQHQQDS^10^ were identified in LPI analysis, indicating that an enrichment of lunasin was achieved during the LPI preparation. In Supplementary Figure S1, the area values for the 2S albumin protein peptides in the PI and LPI samples are shown. The area/intensity value was calculated as the total area/intensity of all peptide features matched to the spectra that identified the peptide. In the lunasin region, there was only one peptide common to both samples. The area values for the peptide QLQGVNLTPC(+57.02)EK, identified in the two samples, were 1.26 × 10^6^ (PI) and 4.27 × 10^6^ (LPI). Thus, the estimated ratio was 3.39.

**Figure 4:**
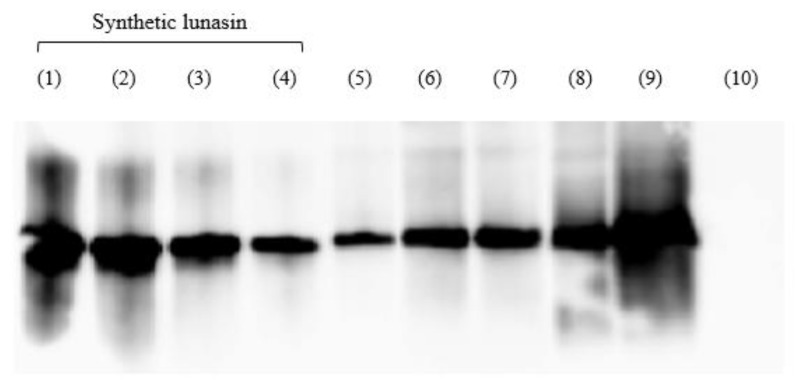
Analysis of soy protein isolate (PI), lunasin-enriched PI (LPI), and its ultrafiltered fractions (LPI-UF1, LPI-UF2, and LPI-UF3) using a Western blot in a PVDF membrane. (1) synthetic lunasin (50 µM), (2) synthetic lunasin (33.33 µM), (3) synthetic lunasin (25 µM), (4) synthetic lunasin (12.5 µM), (5) PI, (6) LPI1 (50 µg of protein), (7) LPI2 (50 µg of protein), (8) LPI-UF1 (50 µg of protein), (9) LPI-UF2 (50 µg of protein), (10) LPI-UF3 (50 µg of protein).

As observed in Figure 4, the presence of lunasin was confirmed in all samples except LPI-UF3, as they showed positive reactivity towards a polyclonal lunasin antibody. Lunasin was mainly retained in the LPI-UF2 fraction due to its molecular weight. The peptide concentration in each sample was calculated by comparing the intensity of each band with the intensity obtained for the synthetic lunasin used as a standard. The results, expressed as mg of lunasin/g of protein and mg of lunasin/g of product are shown in Table 1. The concentration of lunasin for PI was 17.67 mg of lunasin/g of protein, while for LPI the concentration was of 19.45 mg of lunasin/g of protein. This indicates that a slight enrichment of this peptide was achieved with aqueous solvent extraction. These values were in the range (9.2 to 25.7 mg of lunasin/g of protein) reported for different commercial lunasin-enriched products [36] and were slightly higher than the value recently determined in our laboratory in an albumin-enriched extract [21]. From the starting concentration of 19.45 mg of lunasin/g of protein in LPI, the final LPI-UF2 with a lunasin concentration of 58.2 mg of lunasin/g of protein presented a three-fold enrichment of this peptide. A similar enrichment was accomplished after subjecting soy flour to a two-step pilot plant-based ultrafiltration process [36]. Our results indicate that a simple ultrafiltration process would be enough to obtain a lunasin-enriched product with potential health benefits.

### 3.2. Biological Activity of LPI and Its Fractions

#### 3.2.1. Antioxidant Activity in Biochemical Assays

The ORAC and ABTS radical scavenging capacities of LPI and its fractions were determined, and the results are shown in Table 2.

LPI showed low ORAC (0.62 µmol TE/mg of protein) and TEAC (0.11 µmol TE/mg of protein) values. This radical scavenging capacity was lower than the value recently determined in our laboratory for an albumin-enriched extract, which had ORAC and TEAC values of 1.67 and 0.23 µmol TE/mg of protein, respectively [21]. This higher activity could have been due to the soybean varieties used to obtain the extract or the extraction method, which could favor the enrichment of radical scavenging peptides including lunasin. While a slight increase in the antioxidant activity was observed for the fraction containing larger peptides, a potent radical scavenging capacity was measured for fractions containing medium- and low-molecular-weight peptides. Thus, LPI-UF3 showed ORAC and TEAC values of 6.43 and 1.62 µmol TE/mg of protein, respectively. Similarly, low-molecular-weight peptides released from soybean proteins have been reported as the major contributors to the antioxidant activity of soy fractions [37,38,39,40]. Small peptides released from other food sources have also been recognized as the major contributors responsible for their peroxyl radical scavenging properties [41]. Therefore, our results are in agreement with the literature that has reported the high antioxidant activity of small sequences (three to six amino acids) [42], although the contribution of medium-sized peptides such as lunasin cannot be discarded [43,44]. Thus, the LPI-UF2 fraction, which presented the highest lunasin content in the present study, also showed notable antioxidant activity, with ORAC and TEAC values of 2.88 and 0.75 µmol TE/mg of protein, respectively. Thus, our results support the conclusion that, in addition to polyphenols and vitamins, other compounds such as peptides can exert beneficial effects that protect against oxidative stress through their radical scavenging capacity [45].

#### 3.2.2. Protective Effects in RAW 264.7 Macrophage Cells

To evaluate the antioxidant and immunomodulatory effects of soybean proteins and the derived peptides, a macrophage cell model was used under both basal and stimulated conditions. First, the cell viability was tested for LPI and its fractions at concentrations ranging from 0.25 to 1 mg of protein/mL. At these doses, no cytotoxic effects were observed for LPI, LPI-UF1, and LPI-UF2. However, the LPI-UF3 fraction exerted a potent inhibitory effect on cell viability that could have been due to the enrichment of salts resulting from the ultrafiltration process. Thus, this fraction was not further used in the study.

**Figure 5:**
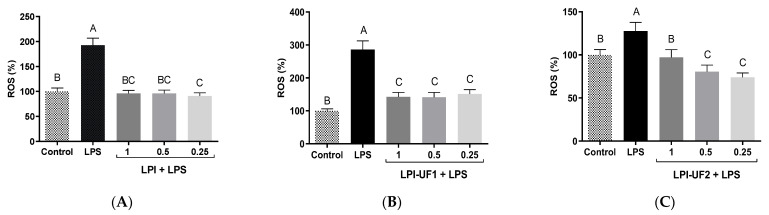
Antioxidant effects of lunasin-enriched soybean protein isolate (LPI) and its ultrafiltered fractions in RAW 264.7 macrophages. Effects of LPI on reactive oxygen species (ROS) production (expressed as %) in RAW 264.7 macrophages stimulated with bacterial lipopolysaccharide (LPS, 100 ng/mL) and treated with (**A**) LPI; (**B**) ˃10 kDa fraction (LPI-UF1); or (**C**) 3–10 kDa fraction (LPI-UF2). Different letters indicate significant differences (*p* ˂ 0.01).

The ROS levels were measured using a DCFH-DA probe in RAW 264.7 cells under LPS-challenging conditions (Figure 5). The intracellular ROS level is recognized as a good indicator of oxidative damage in living cells [46].

In LPS-challenged cells (100 ng/mL), ROS production by macrophages was induced, as previously reported [47]. Both LPI and LPI-UF1 showed potent antioxidant effects, reducing ROS levels up to those shown by the cells under basal conditions, without differences among the tested doses (Figure 5A,B). ROS-reducing effects in this cell model were also observed in our laboratory using an albumin-enriched extract at 7.5 µg of protein/mL [21]. In the case of LPI-UF2, while the highest concentration (1 mg of protein/mL) reduced ROS production (99.81%) up to the levels shown by basal cells (100.00%), the lowest concentrations (0.5 and 0.25 mg of protein/mL) provided higher protection against oxidative damage, reducing ROS levels up to 80.52%, and 74.00%, respectively (Figure 5C). This behavior suggests that at low doses, the antioxidant compounds in the LPI fraction surpassed the potential pro-oxidant activity of the other compounds also present in the fraction. Similarly, low doses of *Erythrina edulis* protein digest and its >10 kDa fraction showed antioxidant activity in this cell model, while high doses showed pro-oxidant effects [48].

The effect of LPI and its ultrafiltered fractions on NO production by RAW 264.7 cells was assessed using the Griess assay. NO is a free radical that is able to react with superoxide anions, producing an oxidant that interacts with and damages cell molecules and membranes, resulting in cell death [49]. However, macrophages produce this compound as an intermediate in their cytotoxic action against pathogens, favoring their phagocytic activity [50].

**Figure 6:**
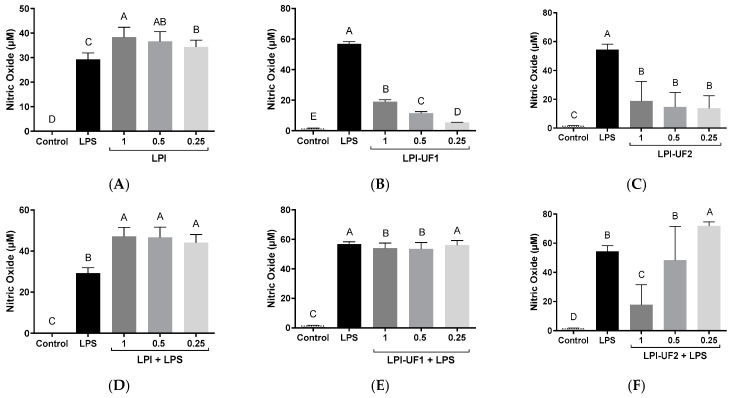
Immunomodulatory effects of lunasin-enriched soybean protein isolate (LPI) and its ultrafiltered fractions on RAW 264.7 macrophages. Effects on nitric oxide (NO, µM) levels in RAW 264.7 macrophages treated with (**A**,**D**) LPI; (**B**,**E**) ˃10 kDa fraction (LPI-UF1), or (**C**,**F**) 3–10 kDa fraction (LPI-UF2) under (**A**–**C**) basal or (**D**–**F**) lipopolysaccharide (LPS)-challenged conditions (60 ng/mL). Different letters indicate significant differences (*p* ˂ 0.01).

Figure 6 summarizes the results obtained by exposing basal and LPS-challenged macrophages to LPI and its ultrafiltered fractions. Although NO production could not be detected in basal conditions, after stimulating the cells with LPS, the levels of this mediator significantly increased (Figure 6A–C). Under basal conditions, LPI (1 mg of protein/mL) induced NO production up to 38.37 µM (Figure 6A). This value was higher than that determined in untreated LPS-challenged cells (29.29 µM). Although lunasin was demonstrated to reduce the NO production induced by LPS in macrophages [51], our previous study also reported NO-inducing effects in this cell model [21].

The disagreement observed between our studies and those using pure lunasin could be due to the concentration of lunasin present in the soybean extracts or the presence of other peptides that are able to induce NO release. In the case of the ultrafiltered fractions, although the treatment of cells with these soybean samples also induced NO release, the highest values that were reached (18.96 and 18.63 µM for LPI-UF1 and LPI-UF2, respectively) were lower than those reached in cells treated with the whole sample (Figure 6B,C). This result suggests that the combined action of high-, medium-, and low-molecular-weight peptides in LPI could be responsible for the higher immunostimulatory effect observed for this sample in comparison with its ultrafiltered fractions. Previous works have also described the NO-inducing properties of peptides from other food sources, although these controversial results preclude the association of immunostimulatory effects with a specific range of molecular weights. Thus, while Li and coworkers [52] reported the ability of the <3 kDa peptide fraction from *Tricholoma matsutake* water extract to induce NO production by basal macrophages, Correa et al. did not find any effect for low-molecular-weight peptides released from pajuro protein under simulated gastrointestinal digestion [48]. The peptides isolated from *Anguilla anguilla* also stimulated NO release by basal RAW 264.7 cells in a dose-dependent way, although the responsible sequences were not identified [53]. However, the sequence YGPSSYGYG was identified as the major sequence responsible for the macrophages activating and the NO-inducing properties of *Pseudostellaria heterophylla* protein hydrolysates [54].

In LPS-challenged cells, LPI potentiated the effects of LPS, reaching NO values of 47.12 µM at a dose of 1 mg of protein/mL, without significant differences among doses (*p* ˂ 0.01, Figure 6D). However, the fraction containing peptides >10 kDa at 1 and 0.5 mg of protein/mL slightly reduced the NO-inducing effect of LPS, while the lowest dose did not provoke any change (Figure 6E). In the case of the fraction containing 3–10 kDa peptides, the dose of 1 mg of protein/mL provoked a potent reduction in the NO induction caused by LPS, decreasing NO levels up to 17.78 µM (Figure 6F). The intermediate dose did not provoke any change, and the lowest dose (0.25 mg of protein/mL) induced the LPS-stimulating action of LPS. Although the presence of the peptide lunasin in this fraction could contribute to the observed NO-reducing effects [51], other peptides with immunostimulatory powers could antagonize the effects of lunasin, resulting in an induction of the immune response by macrophage cells.

## 4. Conclusions

A commercial soybean protein isolate was used as the raw material to produce an extract with radical scavenging properties and antioxidant and immunomodulatory effects in an innate immune cell model. The fractionation process through ultrafiltration achieved the enrichment of the multifunctional peptide lunasin, although multiple other sequences identified by the proteomic analysis could also have contributed to the observed effects. The protective effects against the oxidative stress induced by LPS in the macrophage model could have been mediated by the radical scavenging capacity of peptides present in the soybean samples. Under basal conditions, the extract and its ultrafiltered fractions could activate macrophages, inducing the release of the immune response mediator NO. However, under LPS-induced conditions, the whole extract potentiated the NO-stimulatory effects of LPS, while the fraction containing 3–10 kDa peptides, including lunasin, reduced the increased NO levels resulting from the LPS challenge. The identification of the major contributors responsible for the health benefits and the complete elucidation of the mechanisms of action are needed to confirm the promising role of soybean proteins/peptides as ingredients in functional foods or nutraceuticals that aim to prevent oxidative stress and/or diseases associated with an altered immune response.

## Figures and Tables

**Table 1:** Protein concentration and lunasin peptide concentration (expressed as mg/g of protein and mg/g of product) of soy protein isolate (PI), lunasin-enriched PI (LPI), and its ultrafiltered fractions.

Sample	Protein (%)	Lunasin Concentration	
		mg/g Protein	mg/g Product
Soy Protein Isolate (PI)	75.59	17.67	13.36
Lunasin-enriched Protein Isolate Extract tract (LPI)	35.49	19.45	6.84
LPI-UF1	33.99	23.53	8.00
LPI-UF2	3.10	59.85	1.86
LPI-UF3	1.30	n.d.	n.d.

LPI-UF1: >10 KDa fraction from LPI; LPI-UF2: 3–10 kDa fraction from LPI; LPI-UF3: <3 KDa fraction from LPI; n.d.: not detected.

**Table 2:** ORAC and ABTS radical scavenging capacities (expressed as µmol equivalent of Trolox (TE)/mg of protein) of the lunasin-enriched soy protein isolate extract (LPI) and its ultrafiltered (LPI-UF1, LPI-UF2, and LPI-UF3) fractions.

Sample	ORAC (µmol TE/mg of Protein)	TEAC (µmol TE/mg of Protein)
Soy Protein Isolate Extract (LPI)	0.62 ± 0.04	0.11 ± 0.00
LPI-UF1	0.83 ± 0.02	0.10 ± 0.00
LPI-UF2	2.88 ± 0.05	0.75 ± 0.04
LPI-UF3	6.43 ± 0.29	1.62 ± 0.01

LPI-UF1: >10 KDa fraction from LPI; LPI-UF2: 3–10 kDa fraction from LPI; LPI-UF3: <3 KDa fraction from LPI.

## Data Availability

The data is contained within the manuscript and Supplementary Materials.

## Supplementary Materials

The following supporting information can be downloaded at: https://www.mdpi.com/article/10.3390/plants12102011/s1, Table S1: Mass spectrometry analysis of soy protein isolate (PI); Table S2: Mass spectrometry analysis of lunasin-enriched soy protein isolate (LPI); Figure S1: area values for the 2S albumin protein peptides in the soy protein isolate (PI) and lunasin-enriched soy protein isolate (LPI).
