# Supplementary material for: Contribution of Proteins and Peptides to the Impact of a Soy Protein Isolate on Oxidative Stress and Inflammation-Associated Biomarkers in an Innate Immune Cell Model
Source: Plants (Basel). 2023 May 17;12(10):2011. doi: 10.3390/plants12102011 (PMC10223871; doi:10.3390/plants12102011)
Supplement: Supplementary file 1 [file plants-12-02011-s001.zip › plants-2356561 Figure S1.pdf]

plants

MDPI

Supplementary Figure

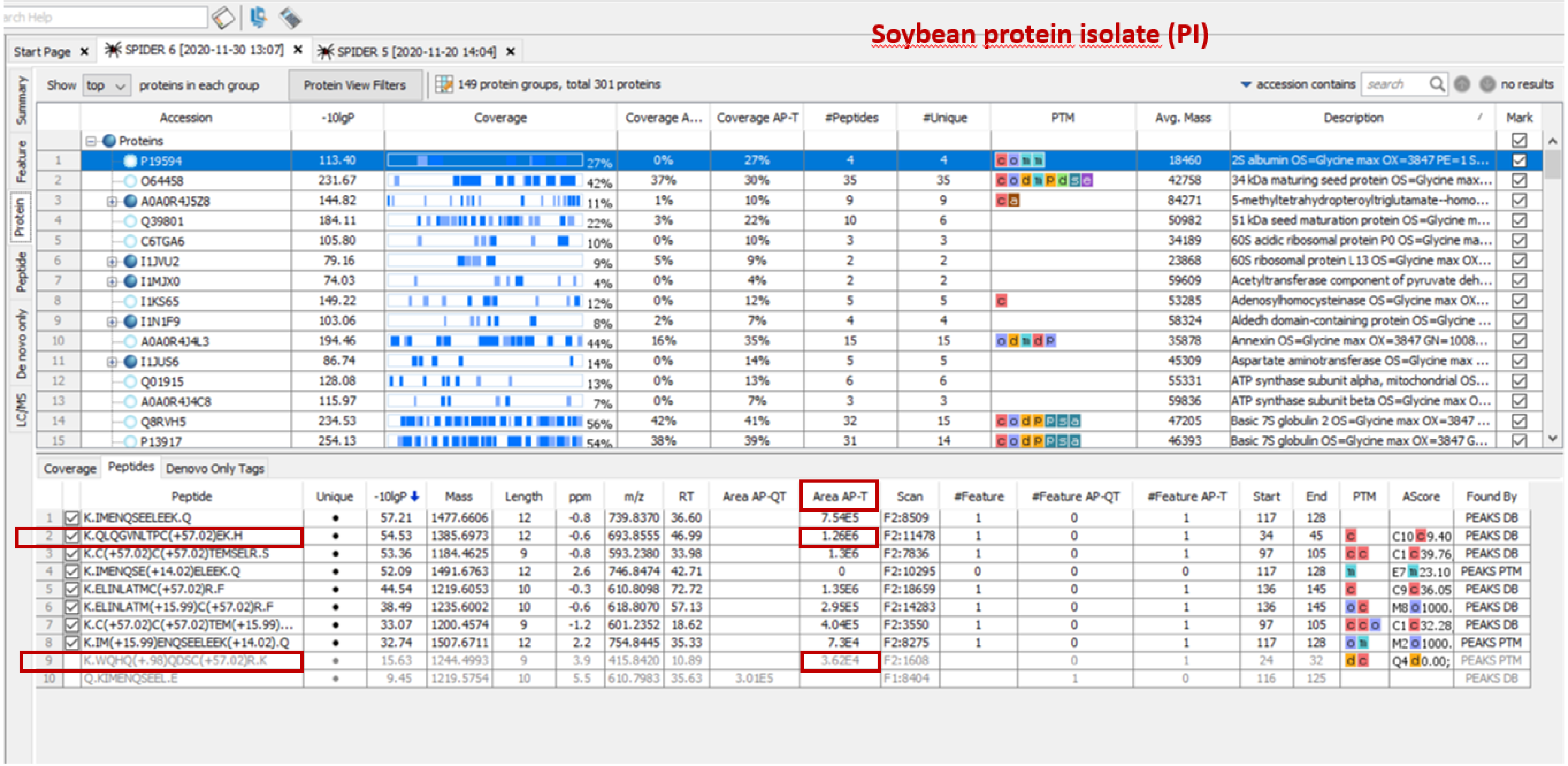

	Accession	-10lgP	Coverage	Coverage A…	Coverage AP-T	#Peptides	#Unique	PTM	Avg. Mass	Description	Mark
	Proteins										☑
1	P19594	113.40	27%	0%	27%	4	4	c o	18460	2S albumin OS=Glycine max OX=3847 PE=1 S…	☑
2	O64458	231.67	42%	37%	30%	35	35	c o d P d	42758	34 kDa maturing seed protein OS=Glycine max…	☑
3	A0A0R4J5Z8	144.82	11%	1%	10%	9	9	c a	84271	5-methyltetrahydropteroyltriglutamate--homo…	☑
4	Q39801	184.11	22%	3%	22%	10	6		50982	51 kDa seed maturation protein OS=Glycine m…	☑
5	C6TGA6	105.80	10%	0%	10%	3	3		34189	60S acidic ribosomal protein P0 OS=Glycine ma…	☑
6	I1JVU2	79.16	9%	5%	9%	2	2		23868	60S ribosomal protein L13 OS=Glycine max OX…	☑
7	I1MJX0	74.03	4%	0%	4%	2	2		59609	Acetyltransferase component of pyruvate deh…	☑
8	I1KS65	149.22	12%	0%	12%	5	5	c	53285	Adenosylhomocysteinase OS=Glycine max OX…	☑
9	I1N1F9	103.06	8%	2%	7%	4	4		58324	Aldedh domain-containing protein OS=Glycine …	☑
10	A0A0R4J4L3	194.46	44%	16%	35%	15	15	o d d P	35878	Annexin OS=Glycine max OX=3847 GN=1008…	☑
11	I1JUS6	86.74	14%	0%	14%	5	5		45309	Aspartate aminotransferase OS=Glycine max …	☑
12	Q01915	128.08	13%	0%	13%	6	6		55331	ATP synthase subunit alpha, mitochondrial OS…	☑
13	A0A0R4J4C8	115.97	7%	0%	7%	3	3		59836	ATP synthase subunit beta OS=Glycine max O…	☑
14	Q8RVH5	234.53	56%	42%	41%	32	15	c o d P P s a	47205	Basic 7S globulin 2 OS=Glycine max OX=3847 …	☑
15	P13917	254.13	54%	38%	39%	31	14	c o d P P s a	46393	Basic 7S globulin OS=Glycine max OX=3847 G…	☑

		Peptide	Unique	-10lgP	Mass	Length	ppm	m/z	RT	Area AP-QT	Area AP-T	Scan	#Feature	#Feature AP-QT	#Feature AP-T	Start	End	PTM	AScore	Found By
1	☑	K.IMENQSEELEEK.Q	•	57.21	1477.6606	12	-0.8	739.8370	36.60		7.54E5	F2:8509	1	0	1	117	128			PEAKS DB
2	☑	K.QLQGVNLTPC(+57.02)EK.H	•	54.53	1385.6973	12	-0.6	693.8555	46.99		1.26E6	F2:11478	1	0	1	34	45	c	C10 c 9.40	PEAKS DB
3	☑	K.C(+57.02)C(+57.02)TEMSELR.S	•	53.36	1184.4625	9	-0.8	593.2380	33.98		1.3E6	F2:7836	1	0	1	97	105	c c	C1 c 39.76	PEAKS DB
4	☑	K.IMENQSE(+14.02)ELEEK.Q	•	52.09	1491.6763	12	2.6	746.8474	42.71		0	F2:10295	0	0	0	117	128		E7 23.10	PEAKS PTM
5	☑	K.ELINLATMC(+57.02)R.F	•	44.54	1219.6053	10	-0.3	610.8098	72.72		1.35E6	F2:18659	1	0	1	136	145	c	C9 c 36.05	PEAKS DB
6	☑	K.ELINLATM(+15.99)C(+57.02)R.F	•	38.49	1235.6002	10	-0.6	618.8070	57.13		2.95E5	F2:14283	1	0	1	136	145	o c	M8 o 1000.	PEAKS DB
7	☑	K.C(+57.02)C(+57.02)TEM(+15.99)…	•	33.07	1200.4574	9	-1.2	601.2352	18.62		4.04E5	F2:3550	1	0	1	97	105	c c o	C1 c 32.28	PEAKS DB
8	☑	K.IM(+15.99)ENQSEELEEK(+14.02).Q	•	32.74	1507.6711	12	2.2	754.8445	35.33		7.3E4	F2:8275	1	0	1	117	128	o	M2 o 1000.	PEAKS PTM
9		K.WQHQ(+.98)QDSC(+57.02)R.K	•	15.63	1244.4993	9	3.9	415.8420	10.89		3.62E4	F2:1608		0	1	24	32	d c	Q4 d 0.00;	PEAKS PTM
10		Q.KIMENQSEEL.E	•	9.45	1219.5754	10	5.5	610.7983	35.63	3.01E5		F1:8404		1	0	116	125			PEAKS DB

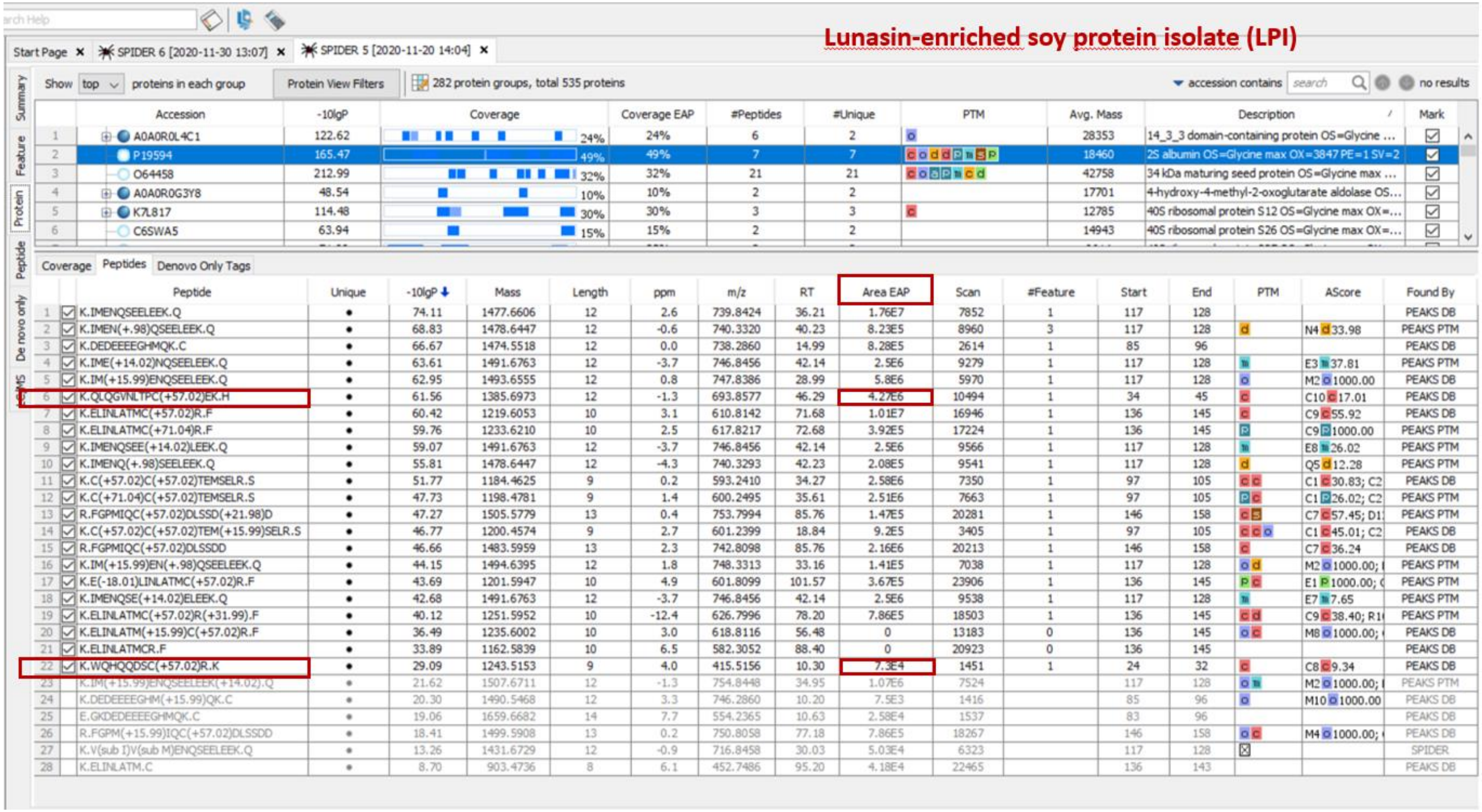

	Accession	-10lgP	Coverage	Coverage EAP	#Peptides	#Unique	PTM	Avg. Mass	Description	Mark
1	A0A0R0L4C1	122.62	24%	24%	6	2	o	28353	14_3_3 domain-containing protein OS=Glycine …	☑
2	P19594	165.47	49%	49%	7	7	c o d d P m S P	18460	2S albumin OS=Glycine max OX=3847 PE=1 SV=2	☑
3	O64458	212.99	32%	32%	21	21	c o a P m c d	42758	34 kDa maturing seed protein OS=Glycine max …	☑
4	A0A0R0G3Y8	48.54	10%	10%	2	2		17701	4-hydroxy-4-methyl-2-oxoglutarate aldolase OS…	☑
5	K7L817	114.48	30%	30%	3	3	c	12785	40S ribosomal protein S12 OS=Glycine max OX=…	☑
6	C6SWA5	63.94	15%	15%	2	2		14943	40S ribosomal protein S26 OS=Glycine max OX=…	☑

	Peptide	Unique	-10lgP	Mass	Length	ppm	m/z	RT	Area EAP	Scan	#Feature	Start	End	PTM	AScore	Found By
1	K.IMENQSEELEEK.Q	•	74.11	1477.6606	12	2.6	739.8424	36.21	1.76E7	7852	1	117	128			PEAKS DB
2	K.IMEN(+.98)QSEELEEK.Q	•	68.83	1478.6447	12	-0.6	740.3320	40.23	8.23E5	8960	3	117	128	d	N4 d 33.98	PEAKS PTM
3	K.DEDEEEEGHMQK.C	•	66.67	1474.5518	12	0.0	738.2860	14.99	8.28E5	2614	1	85	96			PEAKS DB
4	K.IME(+14.02)NQSEELEEK.Q	•	63.61	1491.6763	12	-3.7	746.8456	42.14	2.5E6	9279	1	117	128	m	E3 m 37.81	PEAKS PTM
5	K.IM(+15.99)ENQSEELEEK.Q	•	62.95	1493.6555	12	0.8	747.8386	28.99	5.8E6	5970	1	117	128	o	M2 o 1000.00	PEAKS DB
6	K.QLQGVNLTPC(+57.02)EK.H	•	61.56	1385.6973	12	-1.3	693.8577	46.29	4.27E6	10494	1	34	45	c	C10 c 17.01	PEAKS DB
7	K.ELINLATMC(+57.02)R.F	•	60.42	1219.6053	10	3.1	610.8142	71.68	1.01E7	16946	1	136	145	c	C9 c 55.92	PEAKS DB
8	K.ELINLATMC(+71.04)R.F	•	59.76	1233.6210	10	2.5	617.8217	72.68	3.92E5	17224	1	136	145	P	C9 P 1000.00	PEAKS PTM
9	K.IMENQSEE(+14.02)LEEK.Q	•	59.07	1491.6763	12	-3.7	746.8456	42.14	2.5E6	9566	1	117	128	m	E8 m 26.02	PEAKS PTM
10	K.IMENQ(+.98)SEELEEK.Q	•	55.81	1478.6447	12	-4.3	740.3293	42.23	2.08E5	9541	1	117	128	d	Q5 d 12.28	PEAKS PTM
11	K.C(+57.02)C(+57.02)TEMSELR.S	•	51.77	1184.4625	9	0.2	593.2410	34.27	2.58E6	7350	1	97	105	c c	C1 c 30.83; C2	PEAKS DB
12	K.C(+71.04)C(+57.02)TEMSELR.S	•	47.73	1198.4781	9	1.4	600.2495	35.61	2.51E6	7663	1	97	105	P c	C1 P 26.02; C2	PEAKS PTM
13	R.FGPMIQC(+57.02)DLSSD(+21.98)D	•	47.27	1505.5779	13	0.4	753.7994	85.76	1.47E5	20281	1	146	158	c S	C7 c 57.45; D1	PEAKS PTM
14	K.C(+57.02)C(+57.02)TEM(+15.99)SELR.S	•	46.77	1200.4574	9	2.7	601.2399	18.84	9.2E5	3405	1	97	105	c c o	C1 c 45.01; C2	PEAKS DB
15	R.FGPMIQC(+57.02)DLSSDD	•	46.66	1483.5959	13	2.3	742.8098	85.76	2.16E6	20213	1	146	158	c	C7 c 36.24	PEAKS DB
16	K.IM(+15.99)EN(+.98)QSEELEEK.Q	•	44.15	1494.6395	12	1.8	748.3313	33.16	1.41E5	7038	1	117	128	o d	M2 o 1000.00;	PEAKS PTM
17	K.E(-18.01)LINLATMC(+57.02)R.F	•	43.69	1201.5947	10	4.9	601.8099	101.57	3.67E5	23906	1	136	145	P c	E1 P 1000.00;	PEAKS PTM
18	K.IMENQSE(+14.02)ELEEK.Q	•	42.68	1491.6763	12	-3.7	746.8456	42.14	2.5E6	9538	1	117	128	m	E7 m 7.65	PEAKS PTM
19	K.ELINLATMC(+57.02)R(+31.99).F	•	40.12	1251.5952	10	-12.4	626.7996	78.20	7.86E5	18503	1	136	145	c d	C9 c 38.40; R1	PEAKS PTM
20	K.ELINLATM(+15.99)C(+57.02)R.F	•	36.49	1235.6002	10	3.0	618.8116	56.48	0	13183	0	136	145	o c	M8 o 1000.00;	PEAKS DB
21	K.ELINLATMCR.F	•	33.89	1162.5839	10	6.5	582.3052	88.40	0	20923	0	136	145			PEAKS DB
22	K.WQHQQDSC(+57.02)R.K	•	29.09	1243.5153	9	4.0	415.5156	10.30	7.3E4	1451	1	24	32	c	C8 c 9.34	PEAKS DB
23	K.IM(+15.99)ENQSEELEEK(+14.02).Q	•	21.62	1507.6711	12	-1.3	754.8448	34.95	1.07E6	7524		117	128	o m	M2 o 1000.00;	PEAKS PTM
24	K.DEDEEEEGHM(+15.99)QK.C	•	20.30	1490.5468	12	3.3	746.2860	10.20	7.5E3	1416		85	96	o	M10 o 1000.00	PEAKS DB
25	E.GKDEDEEEEGHMQK.C	•	19.06	1659.6682	14	7.7	554.2365	10.63	2.58E4	1537		83	96			PEAKS DB
26	R.FGPM(+15.99)IQC(+57.02)DLSSDD	•	18.41	1499.5908	13	0.2	750.8058	77.18	7.86E5	18267		146	158	o c	M4 o 1000.00;	PEAKS DB
27	K.V(sub I)V(sub M)ENQSEELEEK.Q	•	13.26	1431.6729	12	-0.9	716.8458	30.03	5.03E4	6323		117	128	☒		SPIDER
28	K.ELINLATM.C	•	8.70	903.4736	8	6.1	452.7486	95.20	4.18E4	22465		136	143			PEAKS DB

Figure S1. Area values for the 2S albumin protein peptides in the soy protein isolate (PI) and lunasin-enriched soy protein isolate (LPI).
